# Insulin‐like growth factor 2 expression in prostate cancer is regulated by promoter‐specific methylation

**DOI:** 10.1002/1878-0261.12164

**Published:** 2018-01-04

**Authors:** Stefan Küffer, Tobias Gutting, Djeda Belharazem, Christian Sauer, Maurice S. Michel, Alexander Marx, Lutz Trojan, Philipp Ströbel

**Affiliations:** ^1^ Institute of Pathology University Medical Center Göttingen University of Göttingen Germany; ^2^ Institute of Pathology University Medical Center Mannheim University of Heidelberg Mannheim Germany; ^3^ Department of Medicine II University Medical Center Mannheim University of Heidelberg Mannheim Germany; ^4^ Department of Urology University Medical Center Mannheim University of Heidelberg Mannheim Germany; ^5^ Department of Urology University Medical Center Göttingen Germany

**Keywords:** IGF2, imprinting, promoter methylation, prostate cancer, targeted therapy

## Abstract

Deregulation of the insulin‐like growth factor (IGF) axis and dysbalance of components of the IGF system as potential therapeutic targets have been described in different tumor types. IGF2 is a major embryonic growth factor and an important activator of IGF signaling. It is regulated by imprinting in a development‐ and tissue‐dependent manner and has been implicated in a broad range of malignancies including prostate cancer (PCa). Loss of imprinting (LOI) usually results in bi‐allelic gene expression and increased levels of IGF2. However, the regulatory mechanisms and the pathophysiological impact of altered IGF2 expression in PCa remain elusive. Here, we show that in contrast to many other tumors, IGF2 mRNA and protein levels were decreased in 80% of PCa in comparison with non‐neoplastic adjacent prostate and were independent of LOI status. Instead, IGF2 expression in both tumors and adjacent prostate depended on preferential usage of the IGF2 promoters P3 and P4. Decreased IGF2 expression in tumors was strongly related to hypermethylation of these two promoters. Methylation of the A region in promoter P4 correlated specifically with IGF2 expression in the 20% of PCa where IGF2 was higher in tumors than in adjacent prostate. We conclude that IGF2 is downregulated in most PCa and may be particularly relevant during early stages of tumor development or during chemotherapy and androgen deprivation. PCa differs from other tumors in that IGF2 expression is mainly regulated through methylation of promoter‐specific and not by imprinting. Targeting of promoter‐specific regions may have relevance for the adjuvant treatment of PCa.

**Abbreviations**
BS6: CTCF‐binding site 6
CTCF: CCCTC‐binding factor
GAPDH: glyceraldehyde‐3‐phosphate dehydrogenase
ICR: imprinting control region
IGF: insulin‐like growth factor
LOI: loss of imprinting
LVI: lymphovascular
PCa: prostate cancer
PIN: prostatic intraepithelial neoplasia
PNI: perineural invasion
PSA: prostate‐specific antigen
RFLP: restriction‐fragment length polymorphisms
ROI: retention of imprinting
SNP: single nucleotide polymorphism

## 1 Introduction

Prostate cancer (PCa) is the most common malignancy in men in the United States and Europe. Nevertheless, knowledge about its pathogenesis and progression is still limited. Age is one of the most important risk factors in PCa. About 75% of all cases occur in men over the age of 65. Therefore, age‐dependent accumulation of DNA damage and epigenetic changes have been proposed to cause this age‐dependent increase (Damaschke *et al*., 2013; Malins *et al*., 2003). Epigenetic changes such as DNA methylation, loss of imprinting (LOI), and histone modification often result in aberrant gene expression that alters cell physiology and may predispose cells to malignant transformation. LOI (i.e., loss of monoallelic expression of specific genes) has been associated with several cancer types and has been suggested to be an early driver of tumorigenesis (Damaschke *et al*., 2013; Hubertus *et al*., 2011; Uribe‐Lewis *et al*., 2011; Xu and Taylor, 2014).

**Figure 1:**
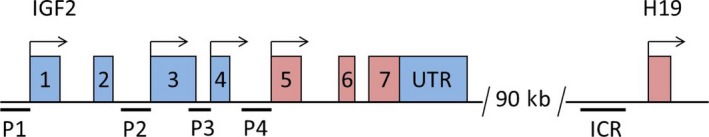
Schematic overview of the IGF2/H19 gene locus with protein coding exons (dark gray) (NG_008849.1) and the four promoter regions P1–P4. The P4 region can be further subdivided into CpG regions P4A, P4B1, and P4B2 (not shown) (Qian *et al*., 2011). The ApaI SNP rs680 is located at the 5′‐UTR. The ICR is located 90 kb downstream of the IGF gene and closer to the H19 locus.

Among the 90 imprinted genes in humans, the *IGF2/H19* (microRNA‐675) gene locus (Fig. 1) is probably the best studied and LOI of *IGF2* is a frequent event in the aging prostate (Fu *et al*., 2008; Kwabi‐Addo *et al*., 2007). Deregulation of the IGF axis is of relevance in a variety of cancers including PCa (Heidegger *et al*., 2015). IGF1 and IGF2 are growth factors that promote cell proliferation, protect from apoptosis, and induce resistance to anticancer therapies through activation of the PI3K‐AKT pathway (Belharazem *et al*., 2016; Hamamura *et al*., 2008).

Several mechanistic models for LOI of *IGF2* have been proposed, among which the most popular (and probably too simplistic) is called the ‘enhancer competition model’ (Nordin *et al*., 2014). According to this model, CCCTC‐binding factor (CTCF), a multizinc finger protein and transcriptional repressor, can bind to an unmethylated imprinting control region (ICR) within the maternal allele of the *IGF2/H19* gene locus and thereby prevents transcription of *IGF2*. On the paternal allele, in contrast, hypermethylation of the ICR prevents CTCF binding and thus allows for *IGF2* expression (Hark *et al*., 2000; Yang *et al*., 2003). To determine the *IGF2* LOI status, we used restriction‐fragment length polymorphisms of a single nucleotide polymorphism (SNP) at *rs680 (820 A/G),* which introduces an ApaI restriction site at *IGF2* exon 7. Cases heterozygous for this SNP at the DNA level can be used to determine whether *IGF2* mRNA is transcribed from one or two alleles (Belharazem *et al*., 2012; Ogawa *et al*., 1993).

Loss of imprinting is believed to result from ICR hypermethylation of both alleles and bi‐allelic transcription of *IGF2*. A good correlation between LOI and increased IGF2 expression in tumors and preneoplastic lesions has been shown for many tumors, including Wilms tumor, colorectal cancer, and esophageal adenocarcinomas (Belharazem *et al*., 2016; Hubertus *et al*., 2011; Mori *et al*., 1996; Nakagawa *et al*., 2001). Bhusari *et al*. (2011) observed LOI of *IGF2* not only in prostatic cancers, but also in adjacent, morphologically normal prostate, suggesting an epigenetic field defect. LOI of *IGF2* in colon mucosa has been associated with an increased cancer risk (Sakatani *et al*., 2005) by enhancing stemness, self‐renewal, and resistance against chemo‐ and radiotherapy (Zhao *et al*., 2016). Very recently, Damaschke *et al*. (2017) described a mouse model with loss‐of‐function mutations of the CTCF‐binding site at the *IGF2‐H19* imprint control region, which resulted in bi‐allelic *IGF2* expression and increased prevalence of prostatic intraepithelial neoplasia (PIN).

However, allelic imprinting is not the only factor that determines IGF2 expression, as its transcription depends also on promoter methylation. The *IGF2* gene contains four promoters (P1–P4), of which P2–P4 are methylated on the paternal allele in young individual (Li *et al*., 1998). The individual transcripts show a tissue‐specific expression pattern during different stages of development (Li *et al*., 1996). While the resulting protein remains unaltered, the distinct transcripts of the four promoters differ in their 5′‐untranslated region and in their translational efficiency and stability (von Horn *et al*., 2002). Previous studies have shown that several regions of these promoters are required for *IGF2* activation in a complex tissue‐ and development‐specific manner (Li *et al*., 1998). In some malignancies, for example, osteosarcoma and hepatocellular carcinoma, promoter hypomethylation was linked to upregulation of *IGF2* mRNA (Li *et al*., 1997, 2009b).

In this study, we investigated the imprinting status, *IGF2* promoter usage, and methylation pattern in correlation to the IGF2 expression in clinical human PCa samples and adjacent, morphologically normal prostate.

## 2 Materials and methods

### 2.1 Prostate patient samples

Frozen prostate samples were collected under stringent quality criteria from 141 patients who underwent radical prostatectomy at the Department of Urology, University Medical Center Mannheim, Germany. The use of PCa specimens for this study was approved by the ethics committee of the University Medical Center Mannheim (2008‐312N‐MA). The mean patient age at surgery was 64.0 years (range: 45–79 years). The mean preresection prostate‐specific antigen (PSA) level was 9.1 ng·mL^−1^ (range: 2.4–58.8 ng·mL^−1^). Eleven tumors were in stage pT2a, *n* = 1 was in stage pT2b, *n* = 67 were in stage pT2c, *n* = 38 were in stage pT3a, *n* = 21 were in stage pT3b, and *n* = 3 were in stage pT4. *n* = 22 tumors had a Gleason score < 7, *n* = 107 tumors had a Gleason score 7 and 8, and *n* = 12 tumors had a Gleason score of > 8 (Table**** 1). Cryosections were HE–stained, and regions of interest were marked by a pathologist (PS). Subsequently, frozen tissue was microdissected to enrich for tumor (hereafter termed ‘T’) and morphologically normal glandular tissue (hereafter termed ‘N’).

**Table 1:** Patient and tumor characteristics

Age	
Mean	64.0
Range	45–79
PSA (ng·mL^−1^)	
Mean (SD)	8.86 (6.39)
Median (range)	6.73 (2–58.8)
Gleason score	
5	5
6	17
7	94
8	13
≥ 9	12
pT stage, *n* (%)	
2a	11 (7.8)
2b	1 (0.7)
2c	67 (47.5)
3a	38 (27)
3b	21 (14.9)
4	3 (2.1)

### 2.2 Isolation of nucleic acid from fresh‐frozen tissue samples

DNA and RNA were isolated from T and adjacent N fresh‐frozen tissue. DNA was isolated using the NucleoSpin Tissue Kit (Macherey‐Nagel, Düren, Germany) according to the manufacturer's protocol and stored at −20 °C. RNA was prepared using TRIzol^®^ reagent (Life Technologies, Waltham, MA, USA) according to the manufacturer's protocol, resuspended in RNase‐free water, and stored at −80 °C.

### 2.3 Extraction of proteins and IGF2 Enzyme‐linked Immunosorbent Assay (ELISA)

Proteins were isolated from fresh‐frozen tissue samples using RIPA buffer containing 1 mm PMSF, 1 mm orthovanadate, and cOmplete™ Protease Inhibitor Cocktail (Roche, Mannheim, Germany). Tissue was minced in lysis buffer and lysed for 45 min on ice. The samples were subsequently centrifuged at high speed for 30 min, and supernatant was recovered. Concentrations of total protein were measured with the DC Protein Assay (Bio‐Rad Laboratories, Hercules, CA, USA) on a microplate reader (Tecan Group Ltd., Switzerland). To measure total IGF2 protein (i.e., free and protein‐bound forms) in tissue samples, we used an IGF2 ELISA kit (Mediagnost, Reutlingen, Germany). The assay was performed according to the manufacturer's protocol; each sample was analyzed in triplicate, and concentrations were calculated according to the standard curve.

### 2.4 IGF2 rs680G>A single nucleotide polymorphism (SNP) genotyping by restriction‐fragment length polymorphism

Analysis of rs680 (G>A) ApaI SNP in the 5′‐UTR of the *IGF2* gene (NG_008849.1) was determined as described previously (Belharazem *et al*., 2012; Ogawa *et al*., 1993). In brief, DNA was amplified using primers F: 5′‐CTTGGACTTTGAGTCAAATTGG‐3′ and R: 5′‐GGTCGTGCCAATTACATTTCA‐3′, followed by HinfI and ApaI restriction digestion. Digestion products were separated on a 2% agarose gel, and results were visually analyzed.

### 2.5 Determination of the imprinting status of the IGF2 gene

The *IGF2* imprinting status was determined by RT‐PCR amplification of RNA from rs680 SNP heterozygous cases and subsequent HinfI and ApaI digestion. Briefly, cDNA was synthetized from 500 ng RNA with the RevertAid™ H Minus First Strand cDNA Synthesis Kit (Thermo Scientific, Waltham, MA, USA) after DNase I treatment (Thermo Scientific). cDNA was amplified by 35 cycles of PCR using the primers F: 5′‐CTTGGACTTTGAGTCAAATTGG‐3′ and R: 5′‐CCTCCTTTGGTCTTACTGGG‐3′. Digestion products were separated on a 2% agarose gel, and results were visually analyzed. A single band after digestion was considered retention of imprinting (ROI), and two bands were considered LOI (Davies, 1993) (Fig. S6).

### 2.6 Relative Quantification of IGF2 and promoter‐specific transcription

Quantitative analyses of IGF2 mRNA and promoter‐specific transcripts were performed on the *Step One Plus Real‐Time PCR‐System* (Life Technologies) using SYBR Green PCR Master Mix (Life Technologies). cDNA was generated as described above. Each sample was analyzed in duplicate. Each run was performed with negative controls using published primers for P1–P4 (Table S1) (Hartmann *et al*., 2001). Relative quantification was computed using the 2^(−ΔΔCT)^ method (Livak and Schmittgen, 2001). Promoter‐specific transcripts from each of the *IGF2* promoters were analyzed by qPCR and statistically tested as described below. Glyceraldehyde‐3‐phosphate dehydrogenase (GAPDH) was used as a reference gene (Table S1).

### 2.7 Analysis of miRNA‐675

TaqMan^®^ MicroRNA Assays (hsa‐miR‐675 and RNU24; Life Technologies) were used to identify specific mature miRNA levels. Briefly, the RT reaction was performed with 10 ng total RNA per reaction. The RT reaction was incubated for 30 min at 16 °C, 30 min at 42 °C, and 5 min at 85 °C; 4 μL of 6× diluted RT product was added to a 10 μL miR‐675/RNU24 assay and Taqman^®^ PCR Mastermix (Life Technologies) reaction. PCRs were performed in technical duplicate on a Step One Plus Real‐Time PCR‐System (Life Technologies) (95 °C for 10 min, followed by 40 cycles of 95 °C for 15 s, and 60 °C for 1 min). After normalization for RNU24, the differences in miR‐675 expression were computed as described above (Livak and Schmittgen, 2001).

### 2.8 Methylation analysis of promoter regions and CTCF‐binding site 6 (BS6)

Methylation of DNA was quantified using the PyroMark™ Q24 system (Qiagen, Hilden, Germany) with corresponding reagents. Bisulfite conversion was performed with the EpiTect Fast Bisulfite Conversion Kit (Qiagen) according to the manufacturer's protocols. In brief, 400 ng of DNA was treated with bisulfite and 10–20 ng of converted DNA was used for the transcript‐specific PCR with SuperHotTaq DNA Polymerase (Bioron, Ludwigshafen, Germany). Primers for promoter methylation analysis were adapted from published methylation‐specific primers (MSP) for P2, P3, and P4 (Qian *et al*., 2011) resulting in primers ‘P2A’, ‘P2B’, ‘P2C1’, ‘P2C2’, ‘P2C3’, for promoter 2, ‘P3’ for promoter 3 and ‘P4A’, ‘P4B1’, ‘P4B2’ for promoter 4 (Table S2). The nine different promoter assays cover 100 CpGs in total. CTCF BS6 (GenBank Accession no. AF087017) spans over 227 nucleotides including 17 CpG islands (Paradowska *et al*., 2009). To cover all CpGs, the BS6 was split into two methylation assays (Table S2). All primers for methylation analysis were designed using PyroMark Assay Design SW 2.0.

### 2.9 Statistical analysis

Correlation analysis between IGF2, H19, and CTCF expression was performed using GraphPad Prism (San Diego, CA, USA) using Spearman's correlation. Fisher's exact test was used to analyze clinical parameters. A *P* value < 0.05 derived from two‐tailed *t*‐test was considered statistically significant. Gaussian distribution was checked using D'Agostino–Pearson normality test.

## 3 Results

### 3.1 IGF2 LOI/ROI is equally distributed in tumors and adjacent prostate

**Figure 2:**
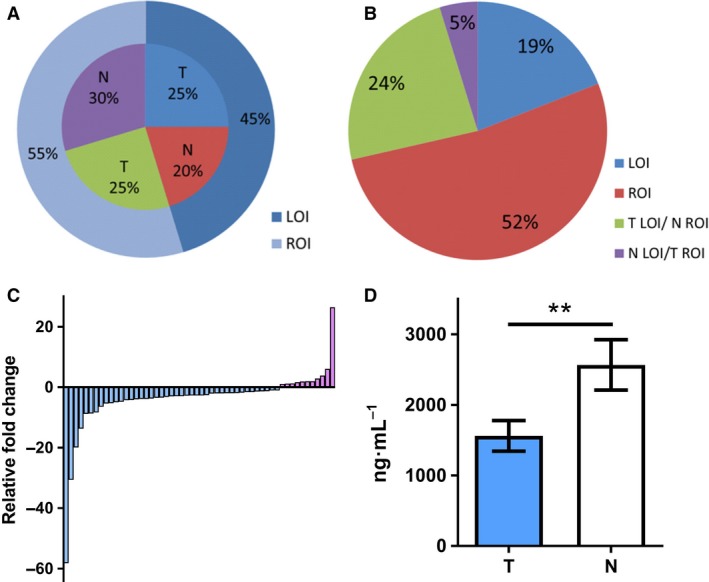
Distribution of imprinting status and IGF2 expression in T and N. (A) Frequency of LOI and ROI in T (*n* = 32) and N (*n* = 32). (B) Concordance of LOI vs. ROI in 22 paired T and N samples. (C) IGF2 mRNA ratio in 60 paired T and N samples. Twelve cases (20%) showed a higher level of IGF2 in T than in N (IGF2high). (D) ELISA measurement of 13 paired samples showed significantly lower IGF2 protein concentration in T than in N (*P* < 0.0012).

As T and N tissue was not available from all 141 patients, 122 N and 128 T (109 paired) samples were screened for IGF2 rs680; 32 N and 32 T (22 paired) samples were heterozygous (i.e., informative) for the ApaI SNP and could be further analyzed for LOI/ROI status (Table 2). Overall, LOI and ROI were equally prevalent in N and T. Among the 64 tested samples, 29 (45%; 16 T [25%] and 13 N [20%]) showed LOI and 35 (55%; 16 T [25%] and 19 N [30%]) showed ROI (Fig. 2A and Table 2). Within the 22 paired samples, 16 (71%) showed concordance with respect to ROI/LOI, six cases (29%) were discordant (five cases LOI in T vs. ROI in N and one case ROI in T and LOI in N) (Fig. 2B).

**Table 2:** Screened patient samples

Patients screened	141
Normal	122
Tumor	128
Paired	109
rs680G>A SNP	
Homozygous (T/N)	76/75
Heterozygous (T/N)	32/32
LOI/ROI (T)	32
LOI/ROI (% LOI)	16/16 (50.0)
LO I/ROI (N)	32
LOI/ROI (% LOI)	13/19 (40.6)
N/T pairs	22
ROI (%)	12 (54.5)
LOI (%)	4 (18.2)
LOI T/ROI N (%)	5 (22.7)
LOI N/ROI T (%)	1 (4.5)

### 3.2 IGF2 mRNA and protein expression are generally lower in tumors than in normal prostate

Overall, the expression of *IGF2* mRNA was significantly higher in N than in T (Fig. S1A). Nevertheless, in 12 cases (20%), the IGF2 mRNA level was higher in T than in N (Fig. 2C). Within this group, paired specimens showed a large variance with fold changes ranging from 1.2 to 26. A similar observation was also found for IGF2 protein using ELISA in 17 paired T and N samples. In 13 paired cases, IGF2 concentrations were significantly lower in T than in N (*P* < 0.0012) (Fig. 2D). Four cases (24%) had higher IGF2 protein concentrations in T than in N, and three of those four also had higher *IGF2* mRNA levels.

### 3.3 Correlation of IGF2 with clinicopathological parameters

*IGF2* expression (either high or low) was not directly correlated with tumor stage, Gleason grade, or PSA level (Fig. S1B–E). However, there was an age‐dependent decrease in IGF2 levels in T compared to N over two decades (*P* < 0.028) (Fig. S1F). In addition, we observed a statistical correlation between *IGF2* imprinting and lymphovascular (LVI) and perineural invasion (PNI): 57% of the cases (four of seven) with LOI showed LVI (vs. 14% in ROI, *P *< 0.025), while PNI was more frequently seen in ROI cases (29%, four of 14 cases with ROI vs. 0%, 0 of 15 cases with LOI, n.s.; Fig. S1G,H).

### 3.4 *IGF2* expression does not correlate with imprinting status and ICR methylation

LOI is thought to result in bi‐allelic *IGF2* transcription and increased IGF2 protein levels. In addition, according to the enhancer competition model, activation of *IGF2* through LOI is accompanied by repression of H19 (miR‐675). We therefore compared the relative *IGF2* mRNA expression to LOI and ROI status and to miR‐675 expression (Fig. S2A–C, D–G, respectively). In N, there was a trend toward higher *IGF2* expression levels in cases with LOI compared to ROI, which was not observed in T. There was no correlation between mRNA expression of *IGF2* and miR‐675/H19 (Fig. S2H,I).

In humans, six CTCF‐binding sites within the ICR of the IGF2/H19 locus have been described with only one of them being relevant for imprinting regulation (Paradowska *et al*., 2009). This region is called the differentially methylated region (DMR). According to the open‐access database JASPAR, (Portales‐Casamar *et al*., 2010) this DMR is coincident with CTCF BS6. To test whether changes in the DMR are relevant for *IGF2* regulation, we investigated methylation at the *CTCF* BS6 locus. There was no difference in the methylation pattern of any of the 17 CpGs within BS6 when comparing N and T in relation to LOI or ROI (Fig. S3).

### 3.5 P3 and P4 promoter usage correlates with IGF2 expression

**Figure 3:**
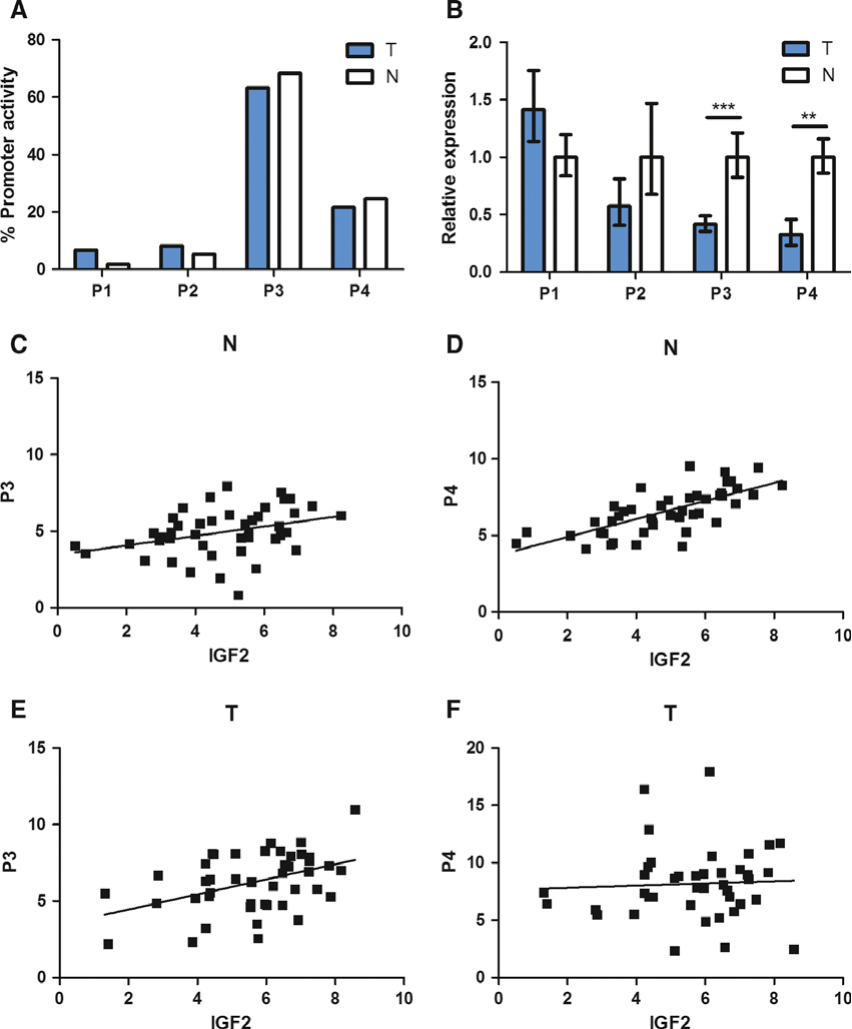
Promoter‐specific IGF2 transcripts (P1–P4) in T and N. (A) Expression of promoter‐specific transcripts P1–P4 in T and N. P3 and P4 were dominant in both T and N. (B) P3‐ and P4‐specific transcripts were significantly higher expressed in N than in T (*P* < 0.0002 and *P* < 0.004, respectively). (C+E) Significant correlation between total IGF2 mRNA and promoter transcripts P3 in T (*r* = 0.42, *P* < 0.009) and N (*r* = 0.45, *P *< 0.002). (D+F) The correlation between total IGF2 mRNA and promoter transcripts P4 was statistically significant only in N (*r* = 0.71, *P* < 0.0001), but not in T (*r* = 0.05295, *P* > 0.05). **P > 0.005, ***P > 0.0005.

As we could not find any clear correlation between LOI and IGF2 expression, we investigated if *IGF2* promoter usage is altered in PCa*. IGF2* is transcribed from four different promoters (P1–P4) in a development‐ and tissue‐specific manner (Qian *et al*., 2011). Therefore, we analyzed the four promoter‐specific transcripts of *IGF2* by qRT‐PCR. In both T and adjacent N, the percentage of transcripts from P3 (68.3%/63.3%) was highest, followed by P4 (24.6%/21.7%), whereas transcripts from P2 (5.3%/8.3%) and P1 (1.8%/6.8%) were much less abundant (Fig. 3A). The relative expression of promoter transcripts P1 and P2 in T and N transcripts was not statistically different, whereas transcripts from P3 and P4 were 2.5‐ to 3‐fold lower in T than in N (Fig. 3B). Overall, the level of P3 and P4 transcripts correlated significantly with IGF2 mRNA levels (Fig. S3E,F). However, when blotting *IGF2* mRNA levels against P3 and P4 expression in T and N separately, we found a significant correlation between IGF2 and both P3 (*r* = 0.41, *P* = 0.0054) and P4 (*r* = 0.75, *P* < 0.0001) in N (Fig. 3C,D), and between IGF2 and P3 (*r* = 0.41, *P* = 0.009), but not P4 (*r* = 0.13) in T (Fig. 3E,F).

### 3.6 Methylation of P3 and P4 is increased in PCa and P4 methylation correlates with high IGF2 expression

**Figure 4:**
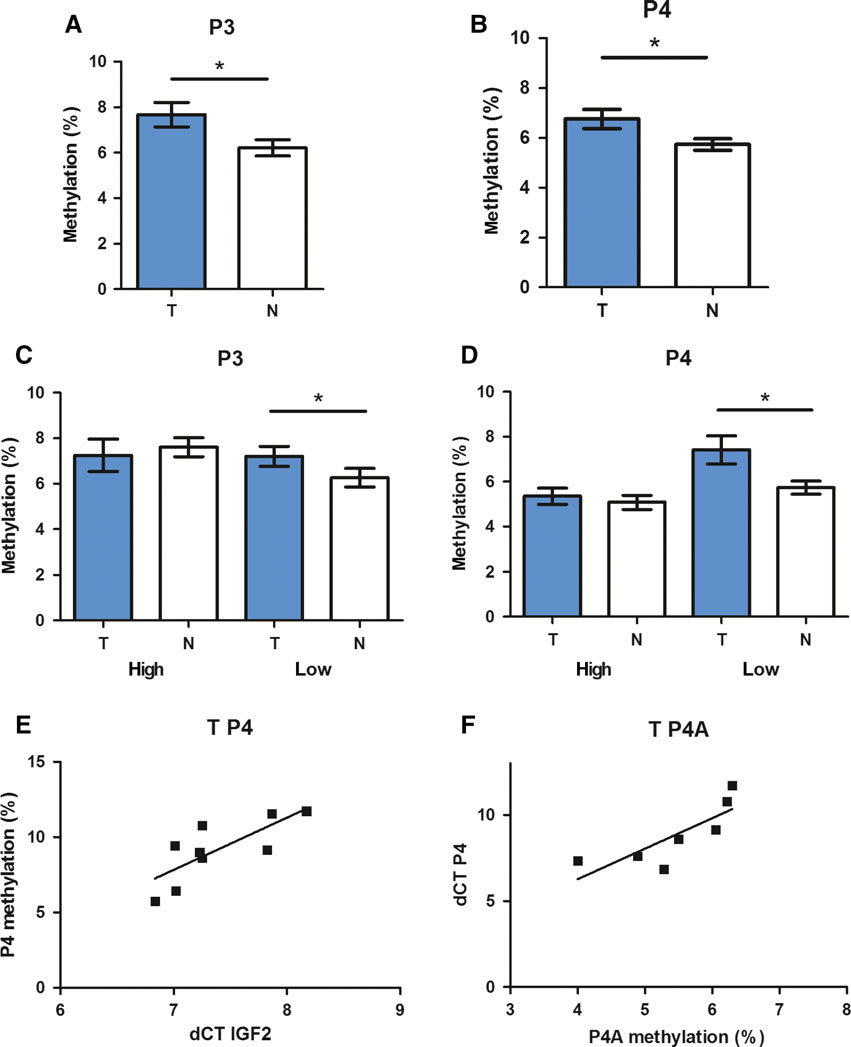
Methylation of promoter P3 and P4 correlates with IGF2 mRNA expression in PCa and normal prostate. (A) Overall higher methylation of P3 (*P* < 0.05) and (B) P4 (*P* < 0.05) in T (*n* = 21) compared to N (*n* = 22). (C+D) However, when cases were separated into IGF2_low_ and IGF2_high_ tumors, this difference was found only in IGF2low tumors for P3 (*P* < 0.05) and P4 (*P *< 0.05). (E) In IGF2_high_ tumors, there was a significant correlation between IGF2 expression and methylation of P4 (*r* = 0.64, *P* < 0.05). (F) The methylation of the CpG in P4A correlated significantly with the P4 promoter‐specific transcript (*r* = 0.80, *P* < 0.05). *P > 0.05.

As only P3 and P4 were found significantly different, we further investigated their methylation status in 16 T and 16 N. The promoter methylation assay was adapted from Qian *et al*. (2011), and CpG methylation of the promoter‐specific regions was quantified by pyrosequencing. P3 and P4 showed a significantly higher methylation in T than in N (Fig. 4A,B), which correlated well with the overall lower IGF2 expression in T.

To investigate if the promoter methylation of P3 and/or P4 is involved in IGF2 regulation, we then separated the T samples into IGF2_high_ cases with elevated and into IGF2_low_ cases with low IGF2 expression. As expected, in IGF2_low_ cases, both promoters P3 and P4 showed higher methylation in T than in paired N samples (Fig. 4C,D). In IGF2_high_ cases, global analysis of the methylation of P3 and P4 promoters revealed no differences between T and N samples. However, P4 methylation in IGF2_high_ was lower than in IGF2_low_ cases. A closer analysis revealed that this difference was mainly due to differential methylation of the first three CPGs within the P4 subregion P4A (Fig. S4). We then analyzed whether P3 and P4 methylation correlates with *IGF2* mRNA expression in T and in the corresponding N of IGF2_high_. *IGF2* expression significantly correlated only with the methylation of P4 in T (Fig. 4E and Fig. S5A–C). To corroborate that the P4 methylation is regulating the P4‐specific transcription, we correlated the P3 and mean P4 methylation with its corresponding promoter‐specific transcripts, but no significance was detected (Fig. S5D, E, H, I). However, a more detailed analysis of the P4 promoter subregions (P4A, P4B1, P4B2) revealed that the CpG methylation of P4A correlated significantly with P4‐specific transcripts of *IGF2* (Fig. 4F, Fig. S5F, G, J–L). This indicates that differential methylation of P4 is responsible for the *IGF2* regulation in T.

## 4 Discussion

Deregulation of the IGF axis in the development of PCa (and other cancers) makes this pathway an interesting candidate for early detection or prevention strategies (Singh *et al*., 2014). The main causes for an intensified IGF signaling are deregulated expression of either IGF‐1R or IGF2 (Li *et al*., 2009a). In this study, we focused on the identification of major factors that govern IGF2 expression in clinical prostate carcinoma samples.

A main observation of this study was the finding that IGF2 expression in PCa, in marked contrast to many other cancers, was reduced in most of the tumors compared to adjacent, morphologically normal tissues. Ribarska *et al*. have reported an overall downregulation of IGF2 and H19 before and suggested ZAC1 as a nodal regulator of the imprinted gene network and as a specific regulator of IGF2. ZAC1 has been described to be downregulated in several cancer types including PCa (Ribarska *et al*., 2014) and might add an additional regulatory level for imprinted genes. Importantly, and in good agreement with observations in normal prostate tissue (Bhusari *et al*., 2011) and in peripheral blood cells of patients with a history of PCa (Belharazem *et al*., 2012), there was a tight correlation between IGF2 levels and imprinting status in normal prostate, which was lost in PCa. These findings suggest that (a) the relevance of imprinting for IGF2 expression varies greatly among different tumor types and (b) that IGF2 imprinting has only a modest impact on IGF2 levels in established human PCas that is overridden by other factors during tumor development. The fact that the prevalence of *IGF2* LOI was similar in tumors and normal prostate and that the *IGF2* imprinting status was often concordant in T and N supports the previous view that *IGF2* LOI is an early event in the development of PCa and indicates an epigenetic field defect in the adjacent ‘normal’ prostatic tissue (Bhusari *et al*., 2011). It has been proposed that LOI of *IGF2* is a common feature of cancer stem cells including those in PCa and that LOI enhances stemness, self‐renewal, and resistance against chemo‐ and radiotherapy (Zhao *et al*., 2016). In good agreement with this concept, Damaschke *et al*. (2017) recently described increased prevalence of PIN in mice with genetically engineered bi‐allelic expression of *IGF2*.

However, in contrast to these early changes in animals, our observations in established human prostatic cancers are not easily compatible with the classic (and oversimplistic) enhancer competition model, as we did not observe a consistent correlation between methylation patterns of the *IGF2* ICR and LOI status, nor with expression of either IGF2 or miR‐675/H19. However, even though *CTCF* may not be the main factor regulating *IGF2* imprinting in PCa, it still remains important by binding recently described regulatory factors like *SUZ12* and vigilin (Liu *et al*., 2014; Wang *et al*., 2015). In colon cancer cells, the polycomb‐repressive complex 2 docking factor *SUZ12* has been proposed to regulate imprinting by binding to the *IGF2* promoter, connecting *CTCF* and the ICR over a long range intrachromosomal loop with *IGF2*. Vigilin has also been shown to build a complex with *CTCF* that binds to the ICR of H19 and contributes to *IGF2/miR‐675/H19* regulation. Furthermore, microRNA may also play a role in *IGF2* imprinting. The oncogenic miR‐483 upregulates *IGF2* expression by binding the promoter P2, hereby reducing in histone H3K27 methylation and decreasing chromatin binding of *CTCF* and *SUZ12* (Zhang *et al*., 2017). In prostate carcinoma samples, we found that IGF2 expression can be best explained by differential *IGF2* promoter usage. Among the four promoters, only P3 and P4 were active and showed significant differences between normal prostate and tumors, whereas P1 and P2 transcripts were not associated with PCa and are probably not relevant for IGF2 expression in adult prostate. In line with observations in other tumor entities (Li *et al*., 2009b; Qian *et al*., 2011), the reduced expression of IGF2 in most PCa compared to normal prostate correlated well with P3 and P4 hypermethylation and with downregulation of the respective IGF2 transcripts. Interestingly, hypomethylation of a specific small CpG region of P4 (P4A) correlated well with upregulation of IGF2 in some PCa. It was recently supposed that the methylation of a single specific CpG is sufficient for transcription factor binding and transcriptional activation and that flanking promoter regions are more activator specific than central parts (Mamrut *et al*., 2013). These findings could indicate an important role of transcription factors in the regulation of IGF2 expression. One of two recently described factors influencing IGF2 expression is the embryonic stem cell transcription factor ZFP57. This factor is overrepresented in cancer and was proposed as a new oncogene (Tada *et al*., 2015). In addition, Lee *et al*. proposed STAT3 as an IGF2 regulating factor. They suggested that STAT3 mediates HDAC inhibitor resistance via direct binding to promoter P3 and P4 and thereby upregulating IGF2. These findings connect IGF2 with advanced disease as STAT3 has been described to promote metastatic progression of PCa (Lee *et al*., 2016). Together with the above‐discussed ZAC1, ZFP57 and STAT3 might be responsible for the increased IGF2 levels in a subset of PCa and may represent imprinting independent regulatory factors with potential therapeutic implications.

While the IGF pathway as a system has recently been shown to be significantly associated with PCa mortality (Cao *et al*., 2014), there was no specific correlation between elevated IGF2 and tumor stage, Gleason score, or PSA levels. However, IGF2 has recently regained considerable interest by the finding that GATA2, a zinc finger transcription factor, mediates chemoresistance via direct upregulation of IGF2 in advanced PCa (Vidal *et al*., 2015). In addition, it has been shown that insulin can upregulate steroid synthesis in cell lines and xenograft tumors in mice (Lubik *et al*., 2011) and that IGF2 can do the same in PCa cells (Lubik *et al*., 2013). Together, these observations could indicate that the relative importance of IGF2 may vary during different stages of a tumor. In PCa, IGF2 seems to be particularly important during early phases of tumorigenesis, or when an advanced tumor is challenged through chemotherapy or androgen deprivation, while it may be less relevant in established tumors under ‘steady‐state’ conditions. A recent publication has linked LOI of IGF2 through increased NFkB signaling as a mechanism to cope with oxidative stress in PCa cells (Yang *et al*., 2014). Thus, it may be relevant to study the regulatory mechanisms (e.g., transcription factor binding) and clinical behavior (e.g., response to androgen deprivation or chemotherapy) in those 20 percentage of PCa with increased IGF2 expression in more detail.

## 5 Conclusions

In summary, we found that IGF2 expression in PCa is independent of the imprinting status of the IGF2‐H19 locus, but rather depends on differential methylation and usage of promoter‐specific. We identified differential methylation of the region 4A within the P4 IGF2 promoter as particularly important for IGF2 levels in PCa. This finding may have relevance for targeting IGF2 to reduce tumor resistance during chemotherapy or androgen deprivation.

## Author contributions

SK, PS contributed to conception and design; PS, AM, CS, MSM, and LT contributed to acquisition of data (acquired and managed patients, provided facilities, etc.); TG, SK, and DB contributed to analysis and interpretation of data (e.g., statistical analysis, biostatistics, computational analysis); SK, PS, and TG wrote the manuscript; SK, TG, DB, AM, MSM, LT, and CS contributed to administrative, technical, or material support (i.e., reporting or organizing data, constructing databases); SK and PS supervised study supervision.

## Supporting information


**Fig. S1.** Correlation of IGF2 expression and imprinting status with clinicopathologic parameters.
**Fig. S2.** Expression and correlation of IGF2 and miR‐675 (H19) in the context of T and N and LOI/ROI.
**Fig. S3.** Methylation analysis of 17 CpGs in binding site 6 (BS6) of CTCF in the ICR.
**Fig. S4.** Differential methylation patterns of promoter P4 regions (P4A, P4B1 and P4B2) in T and N with high and low IGF2 expression.
**Fig. S5.** Comparison of promoter P3‐ and P4‐promoter methylation with IGF2 mRNA in IGF2_high_ samples.
**Fig. S6.** ApaI and Hinf digestion of 14 cDNA amplified PCR‐Products for LOI definition.
**Table S1.** IGF2 and GAPDH Primer.
**Table S2.** Pyrosequencing Primer.

## References

[mol212164-bib-0001] Belharazem D , Kirchner M , Geissler F , Bugert P , Spahn M , Kneitz B , Riedmiller H , Sauer C , Kuffer S , Trojan L *et al* (2012) Relaxed imprinting of IGF2 in peripheral blood cells of patients with a history of prostate cancer. Endocr Connect 1, 87–94. 23781309 10.1530/EC-12-0054 PMC3681323

[mol212164-bib-0002] Belharazem D , Magdeburg J , Berton AK , Beissbarth L , Sauer C , Sticht C , Marx A , Hofheinz R , Post S , Kienle P *et al* (2016) Carcinoma of the colon and rectum with deregulation of insulin‐like growth factor 2 signaling: clinical and molecular implications. J Gastroenterol 51, 971–984. 26984550 10.1007/s00535-016-1181-5

[mol212164-bib-0003] Bhusari S , Yang B , Kueck J , Huang W and Jarrard DF (2011) Insulin‐like growth factor‐2 (IGF2) loss of imprinting marks a field defect within human prostates containing cancer. Prostate 71, 1621–1630. 21432864 10.1002/pros.21379 PMC3825178

[mol212164-bib-0004] Cao Y , Lindstrom S , Schumacher F , Stevens VL , Albanes D , Berndt S , Boeing H , Bueno‐de‐Mesquita HB , Canzian F , Chamosa S *et al* (2014) Insulin‐like growth factor pathway genetic polymorphisms, circulating IGF1 and IGFBP3, and prostate cancer survival. J Natl Cancer Inst 106, dju085. 24824313 10.1093/jnci/dju085 PMC4081624

[mol212164-bib-0005] Damaschke N , Yang B , Bhusari S , Avilla M , Zhong W , Blute ML , Huang W and Jarrard DF (2017) Loss of IGF2 gene imprinting in murine prostate promotes widespread neoplastic growth. Cancer Res 77, 5236–5247. 28775169 10.1158/0008-5472.CAN-16-3089 PMC9741865

[mol212164-bib-0006] Damaschke NA , Yang B , Bhusari S , Svaren JP and Jarrard DF (2013) Epigenetic susceptibility factors for prostate cancer with aging. Prostate 73, 1721–1730. 23999928 10.1002/pros.22716 PMC4237278

[mol212164-bib-0007] Davies SM (1993) Maintenance of genomic imprinting at the IGF2 locus in hepatoblastoma. Cancer Res 53, 4781–4783. 8402661

[mol212164-bib-0008] Fu VX , Dobosy JR , Desotelle JA , Almassi N , Ewald JA , Srinivasan R , Berres M , Svaren J , Weindruch R and Jarrard DF (2008) Aging and cancer‐related loss of insulin‐like growth factor 2 imprinting in the mouse and human prostate. Cancer Res 68, 6797–6802. 18701505 10.1158/0008-5472.CAN-08-1714 PMC4237281

[mol212164-bib-0009] Hamamura K , Zhang P and Yokota H (2008) IGF2‐driven PI3 kinase and TGFbeta signaling pathways in chondrogenesis. Cell Biol Int 32, 1238–1246. 18675921 10.1016/j.cellbi.2008.07.007 PMC2586935

[mol212164-bib-0010] Hark AT , Schoenherr CJ , Katz DJ , Ingram RS , Levorse JM and Tilghman SM (2000) CTCF mediates methylation‐sensitive enhancer‐blocking activity at the H19/Igf2 locus. Nature 405, 486–489. 10839547 10.1038/35013106

[mol212164-bib-0011] Hartmann W , Waha A , Koch A , Albrecht S , Gray SG , Ekstrom TJ , von Schweinitz D and Pietsch T (2001) Promoter‐specific transcription of the IGF2 gene: a novel rapid, non‐radioactive and highly sensitive protocol for mRNA analysis. Virchows Arch 439, 803–807. 11787854 10.1007/s004280100509

[mol212164-bib-0012] Heidegger I , Massoner P , Sampson N and Klocker H (2015) The insulin‐like growth factor (IGF) axis as an anticancer target in prostate cancer. Cancer Lett 367, 113–121. 26231734 10.1016/j.canlet.2015.07.026

[mol212164-bib-0013] von Horn H , Ekstrom C , Ellis E , Olivecrona H , Einarsson C , Tally M and Ekstrom TJ (2002) GH is a regulator of IGF2 promoter‐specific transcription in human liver. J Endocrinol 172, 457–465. 11874694 10.1677/joe.0.1720457

[mol212164-bib-0014] Hubertus J , Lacher M , Rottenkolber M , Muller‐Hocker J , Berger M , Stehr M , von Schweinitz D and Kappler R (2011) Altered expression of imprinted genes in Wilms tumors. Oncol Rep 25, 817–823. 21174059 10.3892/or.2010.1113

[mol212164-bib-0015] Kwabi‐Addo B , Chung W , Shen L , Ittmann M , Wheeler T , Jelinek J and Issa JP (2007) Age‐related DNA methylation changes in normal human prostate tissues. Clin Cancer Res 13, 3796–3802. 17606710 10.1158/1078-0432.CCR-07-0085

[mol212164-bib-0016] Lee SC , Min HY , Jung HJ , Park KH , Hyun SY , Cho J , Woo JK , Kwon SJ , Lee HJ , Johnson FM *et al* (2016) Essential role of insulin‐like growth factor 2 in resistance to histone deacetylase inhibitors. Oncogene 35, 5515–5526. 27086926 10.1038/onc.2016.92 PMC5069101

[mol212164-bib-0017] Li X , Cui H , Sandstedt B , Nordlinder H , Larsson E and Ekstrom TJ (1996) Expression levels of the insulin‐like growth factor‐II gene (IGF2) in the human liver: developmental relationships of the four promoters. J Endocrinol 149, 117–124. 8676043 10.1677/joe.0.1490117

[mol212164-bib-0018] Li X , Gray SG , Flam F , Pietsch T and Ekstrom TJ (1998) Developmental‐dependent DNA methylation of the IGF2 and H19 promoters is correlated to the promoter activities in human liver development. Int J Dev Biol 42, 687–693. 9712523

[mol212164-bib-0019] Li Y , Meng G , Huang L and Guo QN (2009b) Hypomethylation of the P3 promoter is associated with up‐regulation of IGF2 expression in human osteosarcoma. Hum Pathol 40, 1441–1447. 19427670 10.1016/j.humpath.2009.03.003

[mol212164-bib-0020] Li X , Nong Z , Ekstrom C , Larsson E , Nordlinder H , Hofmann WJ , Trautwein C , Odenthal M , Dienes HP , Ekstrom TJ *et al* (1997) Disrupted IGF2 promoter control by silencing of promoter P1 in human hepatocellular carcinoma. Cancer Res 57, 2048–2054. 9158004

[mol212164-bib-0021] Li R , Pourpak A and Morris SW (2009a) Inhibition of the insulin‐like growth factor‐1 receptor (IGF1R) tyrosine kinase as a novel cancer therapy approach. J Med Chem 52, 4981–5004. 19610618 10.1021/jm9002395 PMC2888655

[mol212164-bib-0022] Liu Q , Yang B , Xie X , Wei L , Liu W , Yang W , Ge Y , Zhu Q , Zhang J , Jiang L *et al* (2014) Vigilin interacts with CCCTC‐binding factor (CTCF) and is involved in CTCF‐dependent regulation of the imprinted genes Igf2 and H19. FEBS J 281, 2713–2725. 24725430 10.1111/febs.12816

[mol212164-bib-0023] Livak KJ and Schmittgen TD (2001) Analysis of relative gene expression data using real‐time quantitative PCR and the 2(‐Delta Delta C(T)) Method. Methods 25, 402–408. 11846609 10.1006/meth.2001.1262

[mol212164-bib-0024] Lubik AA , Gunter JH , Hendy SC , Locke JA , Adomat HH , Thompson V , Herington A , Gleave ME , Pollak M and Nelson CC (2011) Insulin increases de novo steroidogenesis in prostate cancer cells. Cancer Res 71, 5754–5764. 21747118 10.1158/0008-5472.CAN-10-2470

[mol212164-bib-0025] Lubik AA , Gunter JH , Hollier BG , Ettinger S , Fazli L , Stylianou N , Hendy SC , Adomat HH , Gleave ME , Pollak M *et al* (2013) IGF2 increases de novo steroidogenesis in prostate cancer cells. Endocr Relat Cancer 20, 173–186. 23319492 10.1530/ERC-12-0250

[mol212164-bib-0026] Malins DC , Johnson PM , Barker EA , Polissar NL , Wheeler TM and Anderson KM (2003) Cancer‐related changes in prostate DNA as men age and early identification of metastasis in primary prostate tumors. Proc Natl Acad Sci U S A 100, 5401–5406. 12702759 10.1073/pnas.0931396100 PMC154357

[mol212164-bib-0027] Mamrut S , Harony H , Sood R , Shahar‐Gold H , Gainer H , Shi YJ , Barki‐Harrington L and Wagner S (2013) DNA methylation of specific CpG sites in the promoter region regulates the transcription of the mouse oxytocin receptor. PLoS One 8, e56869. 23441222 10.1371/journal.pone.0056869 PMC3575498

[mol212164-bib-0028] Mori M , Inoue H , Shiraishi T , Mimori K , Shibuta K , Nakashima H , Mafune K , Tanaka Y , Ueo H , Barnard GF *et al* (1996) Relaxation of insulin‐like growth factor 2 gene imprinting in esophageal cancer. Int J Cancer 68, 441–446. 8945613 10.1002/(SICI)1097-0215(19961115)68:4<441::AID-IJC7>3.0.CO;2-0

[mol212164-bib-0029] Nakagawa H , Chadwick RB , Peltomaki P , Plass C , Nakamura Y and de La Chapelle A (2001) Loss of imprinting of the insulin‐like growth factor II gene occurs by biallelic methylation in a core region of H19‐associated CTCF‐binding sites in colorectal cancer. Proc Natl Acad Sci U S A 98, 591–596. 11120891 10.1073/pnas.011528698 PMC14632

[mol212164-bib-0030] Nordin M , Bergman D , Halje M , Engstrom W and Ward A (2014) Epigenetic regulation of the Igf2/H19 gene cluster. Cell Prolif 47, 189–199. 24738971 10.1111/cpr.12106 PMC6496486

[mol212164-bib-0031] Ogawa O , Eccles MR , Szeto J , McNoe LA , Yun K , Maw MA , Smith PJ and Reeve AE (1993) Relaxation of insulin‐like growth factor II gene imprinting implicated in Wilms’ tumour. Nature 362, 749–751. 8097018 10.1038/362749a0

[mol212164-bib-0032] Paradowska A , Fenic I , Konrad L , Sturm K , Wagenlehner F , Weidner W and Steger K (2009) Aberrant epigenetic modifications in the CTCF binding domain of the IGF2/H19 gene in prostate cancer compared with benign prostate hyperplasia. Int J Oncol 35, 87–96. 19513555 10.3892/ijo_00000316

[mol212164-bib-0033] Portales‐Casamar E , Thongjuea S , Kwon AT , Arenillas D , Zhao X , Valen E , Yusuf D , Lenhard B , Wasserman WW and Sandelin A (2010) JASPAR 2010: the greatly expanded open‐access database of transcription factor binding profiles. Nucleic Acids Res 38, D105–D110. 19906716 10.1093/nar/gkp950 PMC2808906

[mol212164-bib-0034] Qian B , Katsaros D , Lu L , Canuto EM , Benedetto C , Beeghly‐Fadiel A and Yu H (2011) IGF‐II promoter specific methylation and expression in epithelial ovarian cancer and their associations with disease characteristics. Oncol Rep 25, 203–213. 21109978 PMC3075064

[mol212164-bib-0035] Ribarska T , Goering W , Droop J , Bastian KM , Ingenwerth M and Schulz WA (2014) Deregulation of an imprinted gene network in prostate cancer. Epigenetics 9, 704–717. 24513574 10.4161/epi.28006 PMC4063830

[mol212164-bib-0036] Sakatani T , Kaneda A , Iacobuzio‐Donahue CA , Carter MG , de Boom Witzel S , Okano H , Ko MS , Ohlsson R , Longo DL and Feinberg AP (2005) Loss of imprinting of Igf2 alters intestinal maturation and tumorigenesis in mice. Science 307, 1976–1978. 15731405 10.1126/science.1108080

[mol212164-bib-0037] Singh P , Alex JM and Bast F (2014) Insulin receptor (IR) and insulin‐like growth factor receptor 1 (IGF‐1R) signaling systems: novel treatment strategies for cancer. Med Oncol 31, 805. 24338270 10.1007/s12032-013-0805-3

[mol212164-bib-0038] Tada Y , Yamaguchi Y , Kinjo T , Song X , Akagi T , Takamura H , Ohta T , Yokota T and Koide H (2015) The stem cell transcription factor ZFP57 induces IGF2 expression to promote anchorage‐independent growth in cancer cells. Oncogene 34, 752–760. 24469060 10.1038/onc.2013.599

[mol212164-bib-0039] Uribe‐Lewis S , Woodfine K , Stojic L and Murrell A (2011) Molecular mechanisms of genomic imprinting and clinical implications for cancer. Expert Rev Mol Med 13, e2. 21262060 10.1017/S1462399410001717

[mol212164-bib-0040] Vidal SJ , Rodriguez‐Bravo V , Quinn SA , Rodriguez‐Barrueco R , Lujambio A , Williams E , Sun X , de la Iglesia‐Vicente J , Lee A , Readhead B *et al* (2015) A targetable GATA2‐IGF2 axis confers aggressiveness in lethal prostate cancer. Cancer Cell 27, 223–239. 25670080 10.1016/j.ccell.2014.11.013 PMC4356948

[mol212164-bib-0041] Wang H , Ge S , Qian G , Li W , Cui J , Wang G , Hoffman AR and Hu JF (2015) Restoration of IGF2 imprinting by polycomb repressive complex 2 docking factor SUZ12 in colon cancer cells. Exp Cell Res 338, 214–221. 26407907 10.1016/j.yexcr.2015.09.016

[mol212164-bib-0042] Xu Z and Taylor JA (2014) Genome‐wide age‐related DNA methylation changes in blood and other tissues relate to histone modification, expression and cancer. Carcinogenesis 35, 356–364. 24287154 10.1093/carcin/bgt391 PMC3908753

[mol212164-bib-0043] Yang Y , Hu JF , Ulaner GA , Li T , Yao X , Vu TH and Hoffman AR (2003) Epigenetic regulation of Igf2/H19 imprinting at CTCF insulator binding sites. J Cell Biochem 90, 1038–1055. 14624463 10.1002/jcb.10684

[mol212164-bib-0044] Yang B , Wagner J , Damaschke N , Yao T , Wuerzberger‐Davis SM , Lee MH , Svaren J , Miyamoto S and Jarrard DF (2014) A novel pathway links oxidative stress to loss of insulin growth factor‐2 (IGF2) imprinting through NF‐kappaB activation. PLoS One 9, e88052. 24558376 10.1371/journal.pone.0088052 PMC3928145

[mol212164-bib-0045] Zhang Y , Hu JF , Wang H , Cui J , Gao S , Hoffman AR and Li W (2017) CRISPR Cas9‐guided chromatin immunoprecipitation identifies miR483 as an epigenetic modulator of IGF2 imprinting in tumors. Oncotarget 8, 34177–34190. 27486969 10.18632/oncotarget.10918 PMC5470959

[mol212164-bib-0046] Zhao X , Liu X , Wang G , Wen X , Zhang X , Hoffman AR , Li W , Hu JF , Cui J (2016) Loss of insulin‐like growth factor II imprinting is a hallmark associated with enhanced chemo/radiotherapy resistance in cancer stem cells. Oncotarget 7, 51349–51364. 27275535 10.18632/oncotarget.9784 PMC5239480

