# Supplementary material for: Insulin‐like growth factor 2 expression in prostate cancer is regulated by promoter‐specific methylation
Source: Mol Oncol. 2018 Jan 4;12(2):256–66. doi: 10.1002/1878-0261.12164 (PMC5792735; doi:10.1002/1878-0261.12164)
Supplement: Supplementary file 1 — Fig. S1. Correlation of IGF2 expression and imprinting status with clinicopathologic parameters. Fig. S2. Expression and correlation of IGF2 and miR‐675 (H19) in the context of T and N and LOI/ROI. Fig. S3. Methylation analysis of 17 CpGs in binding site 6 (BS6) of CTCF in the ICR. Fig. S4. Differential methylation patterns of promoter P4 regions (P4A, P4B1 and P4B2) in T and N with high and low IGF2 expression. Fig. S5. Comparison of promoter P3‐ and P4‐promoter methylation with IGF2 mRNA in IGF2high samples. Fig. S6. ApaI and Hinf digestion of 14 cDNA amplified PCR‐Products for LOI definition. Table S1. IGF2 and GAPDH Primer. Table S2. Pyrosequencing Primer. [file MOL2-12-256-s001.docx]

Supplement Figure 1. Correlation of IGF2 expression and imprinting status with clinicopathologic parameters. (A) IGF2 expression in 61 paired samples showed an about 2-fold lower expression in T than in N (p=0,0015). No significant differences were detected between LOI (n=17) and ROI (n=20) independent of T and N, LOI (n=7) and ROI (n=9) in T and LOI (n=10) and ROI (n=11) in N. (B) LOI (n=15) and ROI (n=18) in T in correlation to pT stage (%). (C) PSA levels in correlation to LOI (15/13) and ROI (18/20) in T and N respectively. (D) Average Gleason grade in T with LOI (n=15) and ROI (n=17). (E) LOI (15/18) and ROI (13/20) in T and N respectively compared to the average patients age. (F) IGF2 expression in correlation to age. IGF2 levels decrease in comparison to N over 2 decades.  (G) Correlation of *IGF2*imprinting and lymphovascular (LVI) and (H) perineural invasion (PNI): 57% of the cases (4 of 7) with LOI showed LVI (vs. 14% in ROI, p<0.025), while PNI was more frequently seen in in ROI cases (29%, 4 of 14 cases with ROI vs. 0%, 0 of 15 cases with LOI, n.s..

Supplement Figure 2. Expression and correlation of IGF2 and miR-675 (H19) in the context of T and N and LOI/ROI. (A) Correlation between IGF2 and miR-675 in T (n=20). There was no significant correlation detected. (B) Relative expression of IGF2 in LOI (n=17) in comparison to ROI (n=20) independent of tumor state, (C) within N (N LOI (n=10), N ROI (n=11)) and (D) within T (T LOI (n=10),T ROI (n=11)). (E) Relative expression of miR-675 in T (n=30) in comparison to N (n=24), (F) in LOI (12) versus ROI (16) independent of tumor state, (G) within N (N LOI (n=10), (N ROI (n=5)) and (H) within T (nT LOI (n=2), T ROI (n=9)). There was no significant change in any comparison.

Supplement Figure 3. Methylation analysis of 17 CpGs in binding site 6 (BS6) of CTCF in the ICR. (A and B) No significant methylation changes were detected in any CpG comparing T (n=18) and N (n=18) independent of the imprinting status. (C) Single CpG methylation of LOI (n=8) vs ROI (n=16) independent of cancer state and (D) of T (n=18) vs N (n=19). No significant difference was observed. Promoter-specific transcripts of P3 (E) and P4 (F) in correlation to IGF2 mRNA expression. P3 (r=0.48, p<0.0001) and P4 (r=0.31, p=0.0033) transcripts correlated significantly with IGF2 mRNA levels independent of N or T.


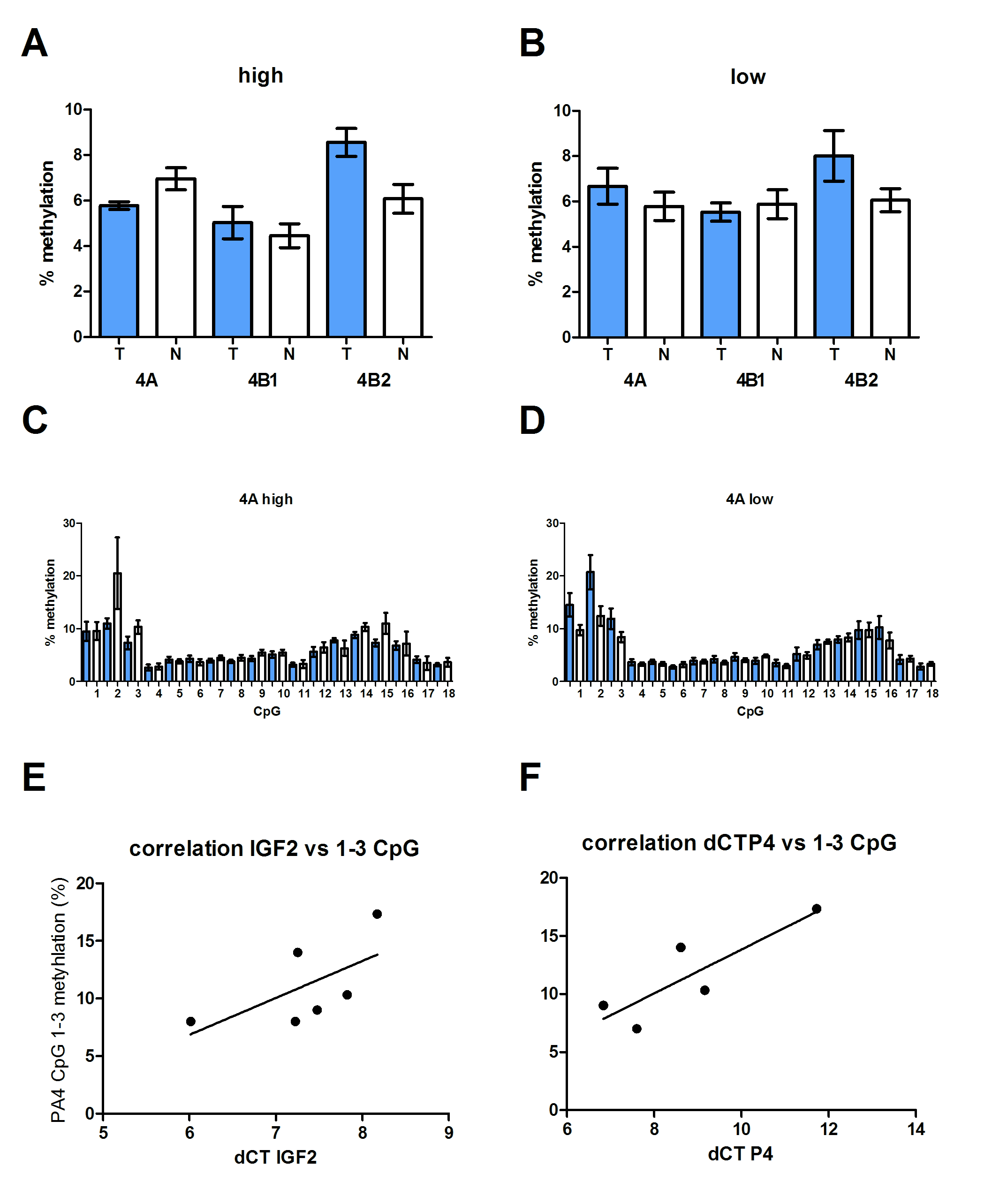


**Supplement Figure 4:** Differential methylation patterns of promoter P4 regions (P4A, P4B1 and P4B2) in T and N with high and low IGF2 expression. The greatest differences between the two groups were found in region P4A. The first three CpGs of P4A showed relative hypermethylation in IGF2 low and relative hypomethylation in IGF2 high tumors.

Supplement Figure 5: Comparison of promoter P3- and P4-promoter methylation with IGF2 mRNA in IGF2_high_ samples. (A and B) The methylation of P3 did not correlate significantly with *IGF2* expression in IGF2_high_ samples (“T high” and “N in T high”) and (C) mean methylation of P4 showed a correlation trend to the *IGF2* expression “N in T high” samples. (D-L) Comparison of promoter (P3 and P4 mean) and individual CpG island cluster methylation (P4 A, P4 B1 and P4 B2) with P3- and P4-promoter specific transcripts in IGF2_high_. Expect of CpG cluster P4 A in T shown in Figure 4F there was no significant correlation detected to promoter specific transcripts.


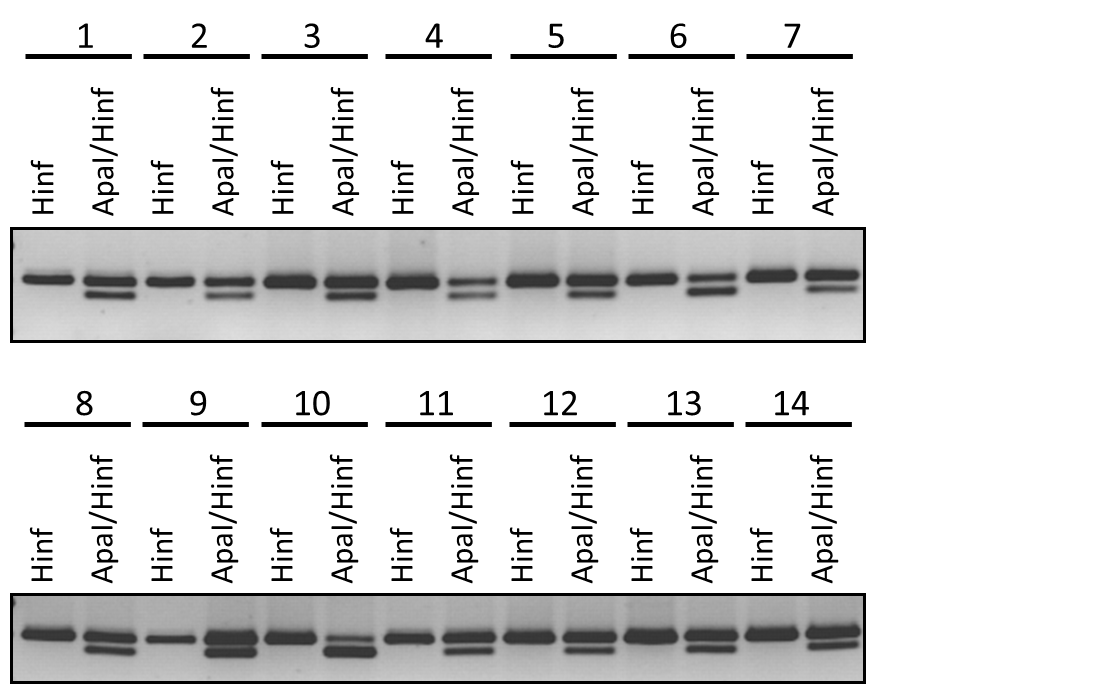


Supplement Figure 6: ApaI and Hinf digestion of 14 cDNA amplified PCR-Products for LOI definition.

Supplement table I: IGF2 and GAPDH Primer

| Primer name | Primer sequence 5’→3’ |
| --- | --- |
| IGF2 LC for | TGGAGACGTACTGTGCTACCC |
| IGF2 LC rev | GGACTGCTTCCAGGTGTCAT |
| GAPDH for | GGTGGTCCAGGGCTCTTACT |
| GAPDH rev | CGACCACTTTGTCAAGCTCA |
| IGF2 P1 for | GCTCCCAGAACTGAGGCTGG |
| IGF2 P2 for | TGCCACAGAGCGTTCGATCG |
| IGF2 P3 for | GCTGTTCGGTTTGCGACACG |
| IGF2 P4 for | GCTTCTCCTGTGAAAGAGAC |
| IGF2 P1-P4 rev | GCGGTAAGCAGCAATGCAGC |

Supplement table II: Pyrosequencing Primer

| Primer name | Primer sequence 5’→ 3’ | Tₐ (°C) |  |
| --- | --- | --- | --- |
| P2A for | AGTTAAGTAGGGGATTAGTTTGTTT* | 55 |  |
| P2A rev | ATTTTTTTTTTTCTTTACCTTCTCCAC |  |  |
| P2A seq | GGTTTAGTTTAGGTT |  |  |
| P2B for | GTTTTTAGTGTGGAGAAGGTAAAGAAA* | 54 |  |
| P2B rev | CCCCCCAAAATCAAAAACTAATTATTAAA |  |  |
| P2B seq | AAATCAAAAACTAATTATTAAACTC |  |  |
| P2C1 for | TTTGGGATTTTTTAAATTTAAGTATTGTT* | 57 |  |
| P2C1 rev | ATCCAAAACCTAAAAAAAAACACATCTA |  |  |
| P2C1 seq | CCAAACCACATAATATAAATCT |  |  |
| P2C2 for | TTTTTAGGTTTTATAGATTTATAGGTTAT | 57 |  |
| P2C2 rev | AATTCCACTTATTTTAAAAACAATACCT* |  |  |
| P2C2 seq | AGGTTTTATAGATTTATAGGTTATT |  |  |
| P2C3 for | GGATGTGGATAGTGTTTTTTTTATATTTAG | 60 |  |
| P2C3 rev | AACCCAACAACAAATATCCCATACTA* |  |  |
| P2C3 seq | AATATTAATTTTAGGGTTTTTTAAG |  |  |
| P3 for | GTAGGTTTTAAGTTTGGTTTAGAT* | 59 |  |
| P3 rev | CCACCTACCCCAACAAAAA |  |  |
| P3 seq | TTAAAACCTCAACCA |  |  |
| P4A for | GTAGGTTTTAAGTTTGGTTTAGAT* | 60 |  |
| P4A rev | CCACCTACCCCAACAAAAA |  |  |
| P4A seq | TTAAAACCTCAACCA |  |  |
| P4B1 for | GTAGAAGTTTATTTTGGTATGTTG | 57 |  |
| P4B1 rev | AAACTCTACCCTTCTTAACCT* |  |  |
| P4B1 seq | AGAAGTTTATTTTGGTATGTTGA |  |  |
| P4B2 for | GGGTTGTTGGTTTGAGGTTAAGAA | 60 |  |
| P4B2 rev | CTCCTTCCCACCTCCTTATAT* |  |  |
| P4B2 seq | GTTAAGAAGGGTAGAGTT |  |  |
| CTCF BS6 1 for | GGGGTTTTTGTATAGTATATGTGTATTT | 59 |  |
| CTCF BS6 1 rev | AACTTAAACTATAATATAAAAACCTACACT* |  |  |
| CTCF BS6 1 seq | TGTATTTTTGGAGGTTTTTT |  |  |
| CTCF BS6 2 for | AGTGTAGGTTTTTATATTATAGTTTAAGT* | 60 |  |
| CTCF BS6 2 rev | TAACTCCCATAAATATTCTATCCCTCACTA |  |  |
| CTCF BS6 2 seq | CTATCCCTCACTACC |  |  |
